# Surgical Methods and Social Factors Are Associated With Long-Term Survival in Follicular Thyroid Carcinoma: Construction and Validation of a Prognostic Model Based on Machine Learning Algorithms

**DOI:** 10.3389/fonc.2022.816427

**Published:** 2022-06-21

**Authors:** Yaqian Mao, Yanling Huang, Lizhen Xu, Jixing Liang, Wei Lin, Huibin Huang, Liantao Li, Junping Wen, Gang Chen

**Affiliations:** ^1^ Shengli Clinical Medical College of Fujian Medical University, Fuzhou, China; ^2^ Department of Endocrinology, Fujian Provincial Hospital, Shengli Clinical Medical College of Fujian Medical University, Fuzhou, China; ^3^ Fujian Provincial Key Laboratory of Medical Analysis, Fujian Academy of Medical, Fuzhou, China

**Keywords:** follicular thyroid carcinoma, machine learning, surgical methods, marital status, prognostic model, AJCC (TNM) staging system

## Abstract

**Background:**

This study aimed to establish and verify an effective machine learning (ML) model to predict the prognosis of follicular thyroid cancer (FTC), and compare it with the eighth edition of the American Joint Committee on Cancer (AJCC) model.

**Methods:**

Kaplan-Meier method and Cox regression model were used to analyze the risk factors of cancer-specific survival (CSS). Propensity-score matching (PSM) was used to adjust the confounding factors of different surgeries. Nine different ML algorithms,including eXtreme Gradient Boosting (XGBoost), Light Gradient Boosting Machine (LightGBM), Random Forests (RF), Logistic Regression (LR), Adaptive Boosting (AdaBoost), Gaussian Naive Bayes (GaussianNB), K-Nearest Neighbor (KNN), Support Vector Machine (SVM) and Multi-Layer Perceptron (MLP),were used to build prognostic models of FTC.10-fold cross-validation and SHapley Additive exPlanations were used to train and visualize the optimal ML model.The AJCC model was built by multivariate Cox regression and visualized through nomogram. The performance of the XGBoost model and AJCC model was mainly assessed using the area under the receiver operating characteristic (AUROC).

**Results:**

Multivariate Cox regression showed that age, surgical methods, marital status, T classification, N classification and M classification were independent risk factors of CSS. Among different surgeries, the prognosis of one-sided thyroid lobectomy plus isthmectomy (LO plus IO) was the best, followed by total thyroidectomy (hazard ratios: One-sided thyroid LO plus IO, 0.086[95% confidence interval (CI),0.025-0.290], *P*<0.001; total thyroidectomy (TT), 0.490[95%CI,0.295-0.814], *P*=0.006). PSM analysis proved that one-sided thyroid LO plus IO, TT, and partial thyroidectomy had no significant differences in long-term prognosis. Our study also revealed that married patients had better prognosis than single, widowed and separated patients (hazard ratios: single, 1.686[95%CI,1.146-2.479], *P*=0.008; widowed, 1.671[95%CI,1.163-2.402], *P*=0.006; separated, 4.306[95%CI,2.039-9.093], *P*<0.001). Among different ML algorithms, the XGBoost model had the best performance, followed by Gaussian NB, RF, LR, MLP, LightGBM, AdaBoost, KNN and SVM. In predicting FTC prognosis, the predictive performance of the XGBoost model was relatively better than the AJCC model (AUROC: 0.886 vs. 0.814).

**Conclusion:**

For high-risk groups, effective surgical methods and well marital status can improve the prognosis of FTC. Compared with the traditional AJCC model, the XGBoost model has relatively better prediction accuracy and clinical usage.

## Introduction

Thyroid carcinoma (TC) is a common endocrine malignant tumor. In recent years, the incidence of TC has been rising sharply worldwide (1, 2). A study from Lim et al. found (3) that between 1974 and 2013, the total incidence of TC in the United States increased by 3% every year. The prognosis of follicular thyroid cancer (FTC) is affected by many factors. However, most current clinical researches focused on papillary thyroid cancer (PTC) and differentiated thyroid cancer (DTC) (4–7), and there is still a lack of large-sample retrospective cohort studies on the prognosis of FTC.

As we all know, surgery is the main method to treat TC, while different surgical methods have different effects on tumor prognosis. On the one hand, there is the possibility of overtreatment. On the other hand, there is the risk of local recurrence caused by conservative surgery. A study by O’Neill etal. (8) revealed that hemithyroidectomy might be the most appropriate treatment for patients with minimally invasive FTC who were younger than 45 years old without vascular invasion. Nixon et al. (9)also confirmed that, for patients with T1T2N0 well differentiated thyroid cancer (WDTC), total thyroidectomy (TT) does not appear to have any benefit in terms of survival compared with patients undergoing thyroid lobectomy. For pT1T2N0 WDTC patients, lobectomy alone is safe and effective (9). On the contrary, a study from Bilimoria et al. (10) indicated that compared with other surgical methods, patients undergoing TT had better survival outcomes and a lower risk of death. However, at present, for the question which surgical method is the best for improving the prognosis of patients, there is still a lack of long-term follow-up study. In recent years, some studies have indicated that sociological factors such as marital status have important impacts on TC (11, 12), but this effect is unclear in patients suffering from FTC only. Other prognostic factors of FTC, such as race, histological type, regional environment, and lymphadenectomy also need to be considered.

With the continuous development of science and technology, artificial intelligence (AI) has been widely used in the medical field. As a branch of AI, machine learning (ML) plays a vital role in disease prevention, screening and diagnosis (13–21). Unfortunately, there is no effective FTC prognostic model based on ML algorithms.The purpose of this study was to review our experience in FTC and assess risk factors for poor prognosis based on initial clinical, sociodemographic and histopathological characteristics. In particular, we aimed to determine whether the FTC patients undergoing only one-sided thyroidlobectomy and isthmectomy (LO plus IO) were sufficient for treatment, explore the relationship between marital status and FTC-specific survival. In the eighth edition of the American Joint Committee on Cancer (AJCC) staging system (22), there are some changes to the TNM staging. However, the role of these new changes in predicting the prognosis of FTC still remains unclear. The ML models were used to predict the prognosis of FTC and compared with the AJCC model. The data for our study came from the database of Surveillance, Epidemiology, and End Results (SEER) and are maintained by the American cancer institute. The SEER database accumulates the survival and prognosis of a large number of rare tumors through long-term follow-up, which provides a valuable opportunity to analyze the prognosis of FTC.

## Patients and Methods

### Data Sources and Study Population

**Figure 1:**
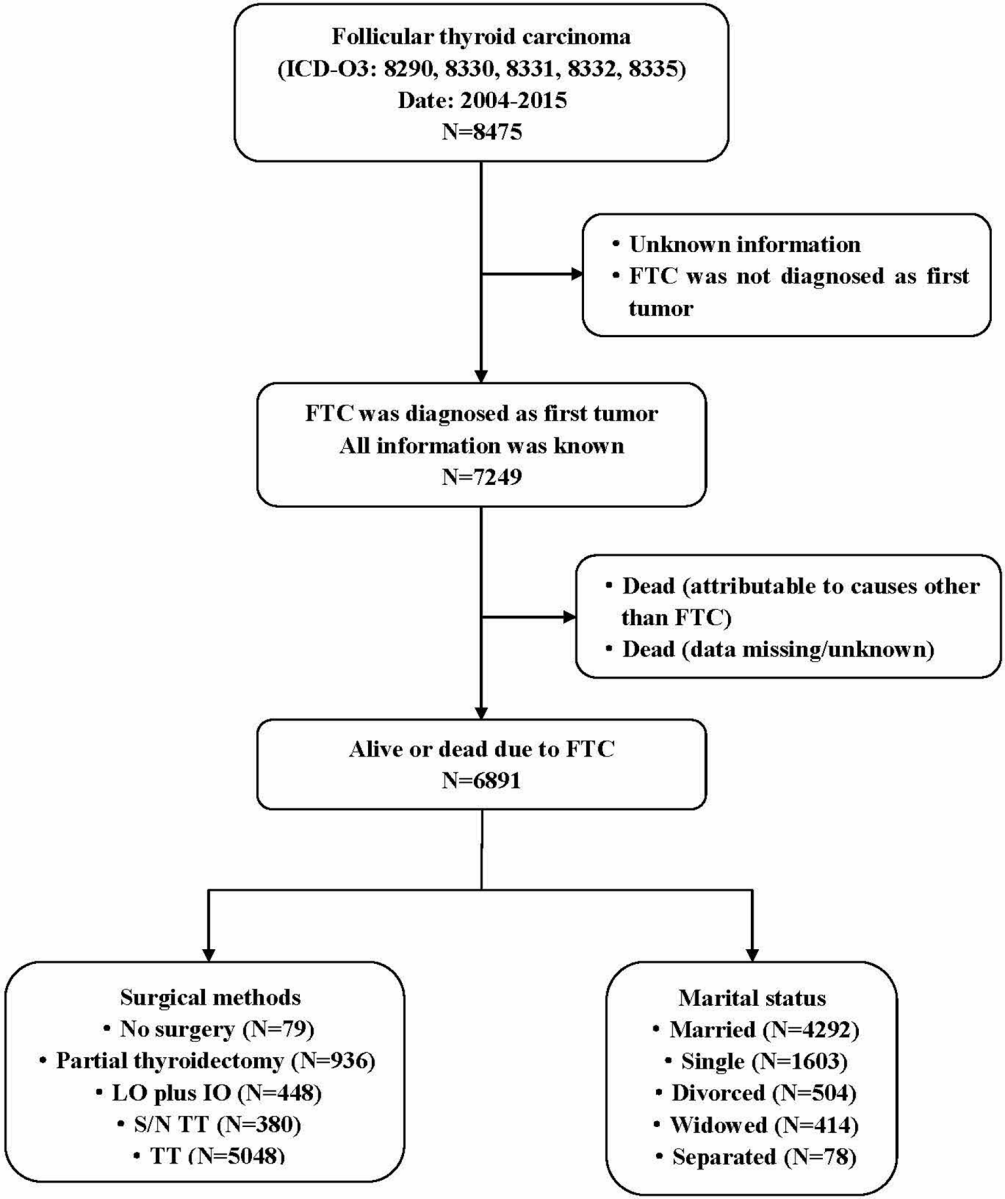
Flow diagram of study design. The data from 6891 patients diagnosed with primary FTC between 2004 and 2015 were included in the study. The study met the Consolidated Standards of Reporting Trials (CONSORT) diagram detailing the study inclusion criteria. Partial thyroidectomy include one-sided thyroid lobectomy or lesion resection. FTC, Follicular thyroid carcinoma; LO plus IO, Lobectomy plus isthmectomy; S/N TT, Subtotal or near total thyroidectomy; TT, Total thyroidectomy.

The data were obtained from the SEER database that is also named “Incidence-SEER 18 Regs Research Data + Hurricane Katrina Impacted Louisiana Cases (1973–2015)”. SEER*Stat 8.3.5 software was used for data acquisition. The information of the SEER database comes from 21 cancer registries and covers more than 28% of cases in the United States (https://seer.cancer.gov/). The subjects of the study were patients who were diagnosed with FTC from 2004 to 2015 in 18 regions of the United States and they were included in the SEER database.It should be noted that the relevant information such as tumor size and degree of capsular invasion was not included in the database until 2004, so the time range of our study was selected from 2004 to 2015. Inclusion criteria: ① There was no restriction on age and gender. ② The histological type was FTC. Exclusion criteria: ① Unknown information/not applicable. ② FTC was not diagnosed as first tumor. ③ FTC was not the main cause of death. The detailed research process was shown in **Figure 1**. The study was deemed to be exempt from formal review, because it used publicly available and confirmed data and gave up the informed consent that was approved by the relevant institutional review board.

### Data Selection and Definition

Based on a large amount of literature reading and expert knowledge, the research variables related to the prognosis of FTC were determined. According to the SEER usage guidelines and the Collaborative Stage Data Collection System (CS Manual Online Help: https://web2.facs.org/cstage202/thyroid/Thyroidschema.html), the information in the SEER database was extracted. In this study, a total of eleven variables closely related to FTC prognosis were included. Variables include sex, age at diagnosis, race, marital status, histological type, region, surgical methods, lymphadenectomy, T classification, N classification, and M classification.

The definition and classification criteria of FTC and its subtypes refer to the histology codes from International Classification of Diseases for Oncology, Third Revision (ICD-O-3) published by the World Health Organization (WHO) in 2008. FTC includes common subtype (8330), oxyphilic variant (8290), well differentiated subtype (8331), trabecular variant (8332), and minimally invasive subtype (8335). FTC was divided into two major categories based on the histological characteristics of tumors: Classic subtype (8330, 8331, 8332, 8335) and oxyphilic variant (8290). It should be noted that the fourth edition of the WHO new pathological classification of thyroid tumors in 2017 reclassified Hürthle cell carcinoma (HCC)/oxyphilic variant as an independent disease type. At present, the clinical, pathological and molecular characteristics of HCC and FTC are still controversial, and there is a lack of large-scale tumor prognosis cohort studies. Therefore, in this study, HCC was still used as an independent subtype of FTC for prognostic analysis. According to the treatment methods, surgical methods were divided into five categories: no surgery on the primary site, partial thyroidectomy (lobectomy or lesion resection), one-sided thyroid LO plus IO, subtotal or near total thyroidectomy (S/N TT), and TT. Marital status was classified on the basis of the status at diagnosis but not specified. Marital status was divided into married, widowed, separated, divorced, and single (unmarried) status. Lymph node dissection was divided into three categories: no lymph node dissection, one to three regional lymph nodes dissection, and four or more regional lymph nodes dissection. According to the eighth edition of the AJCC cancer staging guidelines (22), age at diagnosis and TNM staging were classified. The patient’s attribution area was divided into East, Pacific Coast, Northern Plains, and Southwest in the United States based on the region where the patient’s tumor was registered. The races were divided into three categories, namely white, black and other. Other races include American indian, Alaska native, Asian or Pacific islander. The data were removed from the cohort with missing original information and data that were not statistically significant due to the small sample size.The extraction, definition, and classification of the data were completed by two collaborators (Yaqian Mao and Yanling Huang), and the resulting differences were resolved through discussion.

### Feature Selection and Model Construction

Univariate and multivariate survival analysis were assessed by Cox proportional-hazards model. The proportional hazards assumption was evaluated by schoenfeld residuals (23). Based on the results of multivariate survival analysis, nine commonly used ML algorithms in the medical were chosen to construct prognostic models for FTC. The end point was the patient’s survival status (ie, survival or death) at the end of the 143-month follow-up. The nine ML classifiers include eXtreme Gradient Boosting (XGBoost), Light Gradient Boosting Machine (LightGBM), Random Forests (RF), Logistic Regression (LR), Adaptive Boosting (AdaBoost), Gaussian Naive Bayes (Gaussian NB), K-Nearest Neighbor (KNN), Support Vector Machine (SVM) and Multi-Layer Perceptron (MLP). The SHapley Additive exPlanations (SHAP) method was used to explain the visualization of the model. The goal of SHAP is to explain the prediction of ML by calculating the contribution of each feature to the prediction result, and it is also the most commonly used black box model interpretation method at present (24, 25). The AJCC model was built by the multivariate COX regression analysis, and the R package, named “rms”, “foreign”, “survival” and “survivalROC”, were used to calculate the AUROC value and draw the nomogram and calibration curve.

As an integrated learning algorithm, XGBoost combines the predictions from an ensemble of weak regression trees that are added sequentially to the model to maximize predictive performance and minimize model complexity (26). At the same time, XGBoost adds a complexity control model and learns from RF to reduce the calculation, thus making the model not easy to be over-fitting.As a Gradient Boosted Decision Tree (GBDT) algorithm (27), LightGBM uses a histogram-based algorithm to speed up the training process, reduces memory consumption, and combines advanced network communication to optimize parallel learning that is called the parallel voting decision tree algorithm. RF, an ensemble learning algorithm, is a combination recognition model formed by combining multiple decision trees (28, 29). The accuracy of RF classification is relatively high, it is not easy to be over-fitting, and the anti-noise ability is strong, which is easy to implement, but the amount of calculation is relatively large. NB estimates the conditional probability of each category under each feature by assuming that *P* (x/yi) obeys Gaussian distribution (ie, normal distribution). The NB classifier is widely used in many classification tasks, because its performance is comparable to state-of-the-art classifiers, and it is simple to implement and fast to execute (30, 31). The advantage of the Gaussian NB model is that it has a stable classification efficiency and a relatively simple algorithm, and performs well on small-scale data. LR is one of the most commonly used binary classification algorithms, and is the gold standard for analyzing binary classification medical data (32, 33), because it can not only provide prediction results, but also provide additional information about the prediction results, such as the odds ratio (OR) of the diagnosis and the 95% confidence interval (CI) (34). AdaBoost is a typical boosting algorithm. Using “reweighting”, that is, in each round of the training process, each training sample is provided a new weight according to the sample distribution. By reducing the classification error of individual learner each time, the importance of good individual learner is increased, and the final integrated learner is obtained (35). MLP is a forward structure of artificial neural network (ANN) that is generalized by perceptron. It integrates the neuron model in the perceptron algorithm and overcomes the weakness of the perceptron to recognize linearly inseparable data, and it has the ability to quickly solve complex problems. The ML approach of MLP-ANN is derived from the basic structure of artificial neurons, and the function of the network depends on the training they receive. This training is based on the presentation of real-world examples and simulates the learning process of a system by determining the differences between the response given by the network and the expected behavior (36, 37). KNN means that in the feature space, if most of the k nearest (ie nearest neighbors in the feature space) samples near a sample belong to a certain category, the sample also belongs to this category (38). The advantages of KNN model are high accurate and insensitive to outliers, and no data input assumptions. SVM, an efficient way to build classifiers, aims to create a decision boundary between two classes, thus making it possible to predict labels from one or more feature vectors (39). Combining multiple parameter values, using the SVM classification algorithm in a nonlinear space enables efficient data classification. Compared with other ML methods, SVM is very powerful in identifying subtle patterns in complex datasets, which can be used for tumor prediction (40), genetic screening (41), and drug applications (42, 43).

Resampling method was used to train and test ML classifiers. Model performance evaluation was mainly conducted through the area under the receiver operating characteristic curve (AUROC), accuracy, sensitivity, specificity, and negative predictive value (NPV). Among them, the classifier with the largest AUROC value was selected as the best model.Then, the optimal model was trained through 10-fold cross-validation,so as to improve its prediction accuracy and applicability. The following packages of Python were used for ML model construction and optimization, including “sklearn.linear model”, “sklearn.ensemble”, “xgboost1.2.1”, “lightgbm 3.2.1”, “sklearn 0.22.1”, “shap 0.39.0”, etc.

### Statistical Methods

All statistical analyses in our study were performed using the IBM SPSS software (version 25.0 for windows, SPSS Inc., Chicago, IL, USA), R software (version 3.6.3, https://www.r-project.org/) and Python software (version 3.6.13, https://www.python.org/). In the baseline analysis, categorical variables were represented by counts and proportions, and differences between groups were analyzed using Pearson chi-square tests. In order to reduce the model error caused by the mutual influence between variables, correlation analysis on the data was carried out and showed by heat map. In addition, the variance inflation factor (VIF) was also used to assess the multicollinearity between variables. The relationship between significant variables and cancer-specific survival (CSS) was calculated using the Kaplan-Meier method, and the log-rank test was used to compare distribution differences. CSS was calculated with the cumulative incidence. In order to further adjust the potential bias in our cohort, the propensity score matching (PSM) method was used to match one-sided thyroid LO plus IO with other surgical methods and non-surgical cases. The PSM method is a statistical method for matching the treatment group and the control group, so that the clinical indicators of the research object are comparable to balance variables and reduce bias (44). All statistical analysis adopted two-sided test, and *P* values less than 0.05 indicated significant.

## Results

### Baseline Characteristics

A total of 6891 FTC patients were included in this study, including 4930 female patients and 1961 male patients, with a median follow-up time of 64 months (range, 29 to100 months). The baseline characteristics of all FTC patients were shown in **Table 1**, and the detailed research flowchart was shown in **Figure 1**.

**Table 1:** Demographic characteristics of the participants.

Characteristics	Total 6891	Survival 6650	Death 241	X^2^	*P* -Value
**Age** (y), No.(%)				379.980	<0.001
<25	396 (5.747)	394 (5.925)	2 (0.830)		
25-40	1444 (20.955)	1435 (21.579)	9 (3.734)		
40-55	2244 (32.564)	2210 (33.233)	34 (14.108)		
55~70	1890 (27.427)	1815 (27.293)	75 (31.120)		
70~85	851 (12.349)	749 (11.263)	102 (42.324)		
≥85	66 (0.958)	47 (0.707)	19 (7.884)		
**Sex**, No.(%)				13.644	<0.001
Female	4930 (71.543)	4783 (71.925)	147 (60.996)		
Male	1961 (28.457)	1867 (28.075)	94 (39.004)		
**Race**, No.(%)				2.406	0.300
White	5488 (79.640)	5304 (79.759)	184 (76.349)		
Black	786 (11.406)	757 (11.383)	29 (12.033)		
Other*	617 (8.954)	589 (8.857)	28 (11.618)		
**Marital status**, No.(%)				146.498	<0.001
Married	4292 (62.284)	4176 (62.797)	116 (48.133)		
Single	1603 (23.262)	1562 (23.489)	41 (17.012)		
Divorced	504 (7.314)	484 (7.278)	20 (8.299)		
Widowed	414 (6.008)	358 (5.383)	56 (23.237)		
Separated	78 (1.132)	70 (1.053)	8 (3.320)		
**Region**, No.(%)				0.928	0.819
East	2863 (41.547)	2769 (41.639)	94 (39.004)		
Pacific Coast	3076 (44.638)	2966 (44.602)	110 (45.643)		
Northern Plains	575 (8.344)	552 (8.301)	23 (9.544)		
Southwest	377 (5.471)	363 (5.459)	14 (5.809)		
**Histology**, No.(%)				10.652	0.001
Classic subtype	4905 (71.180)	4756 (71.519)	149 (61.826)		
HCC/Oxyphilic variant	1986 (28.820)	1894 (28.481)	92 (38.174)		
**Surgical methods**, No.(%)				383.321	<0.001
No surgery	79 (1.146)	45 (0.677)	34 (14.108)		
Partial thyroidectomy	936 (13.583)	916 (13.774)	20 (8.299)		
One-sided thyroid LO plus IO	448 (6.501)	445 (6.692)	3 (1.245)		
S/N TT	380 (5.514)	363 (5.459)	17 (7.054)		
TT	5048 (73.255)	4881 (73.398)	167 (69.295)		
**Lymphadenectomy**, No.(%)				24.013	<0.001
None	4871 (70.686)	4717 (70.932)	154 (63.900)		
1 to 3 regional lymph nodes	1460 (21.187)	1413 (21.248)	47 (19.502)		
4 or more regional lymph nodes	560 (8.127)	520 (7.820)	40 (16.598)		
**T classification**, No.(%)				901.804	<0.001
T1	1635 (23.727)	1618 (24.331)	17 (7.054)		
T2	2772 (40.226)	2737 (41.158)	35 (14.523)		
T3	2299 (33.362)	2188 (32.902)	111 (46.058)		
T4	185 (2.685)	107 (1.609)	78 (32.365)		
**N classification**, No.(%)				522.532	<0.001
N0	6687 (97.040)	6506 (97.835)	181 (75.104)		
N1a	110 (1.596)	91 (1.368)	19 (7.884)		
N1b	94 (1.364)	53 (0.797)	41 (17.012)		
**M classification**, No.(%)				1344.483	<0.001
M0	6667 (96.749)	6533 (98.241)	134 (55.602)		
M1	224 (3.251)	117 (1.759)	107 (44.398)		
**AJCC 8th Edition**, No.(%)				1680.481	<0.001
I	5585 (81.048)	5531 (83.173)	54 (22.407)		
II	1035 (15.020)	974 (14.647)	61 (25.311)		
III	44 (0.639)	33 (0.496)	11 (4.564)		
IVa	45 (0.653)	23 (0.346)	22 (9.129)		
IVb	182 (2.641)	89 (1.338)	93 (38.589)		

*Other include American indian/Alaska native, Asian or Pacific islander; Partial thyroidectomy include lobectomy or lesion resection.

HCC, Hürthle cell carcinoma; LO plus IO, Lobectomy plus isthmectomy; S/N TT, Subtotal or near total thyroidectomy; TT, Total thyroidectomy; AJCC, American Joint Committee on Cancer.

### Feature Variable Screening

**Figure 2:**
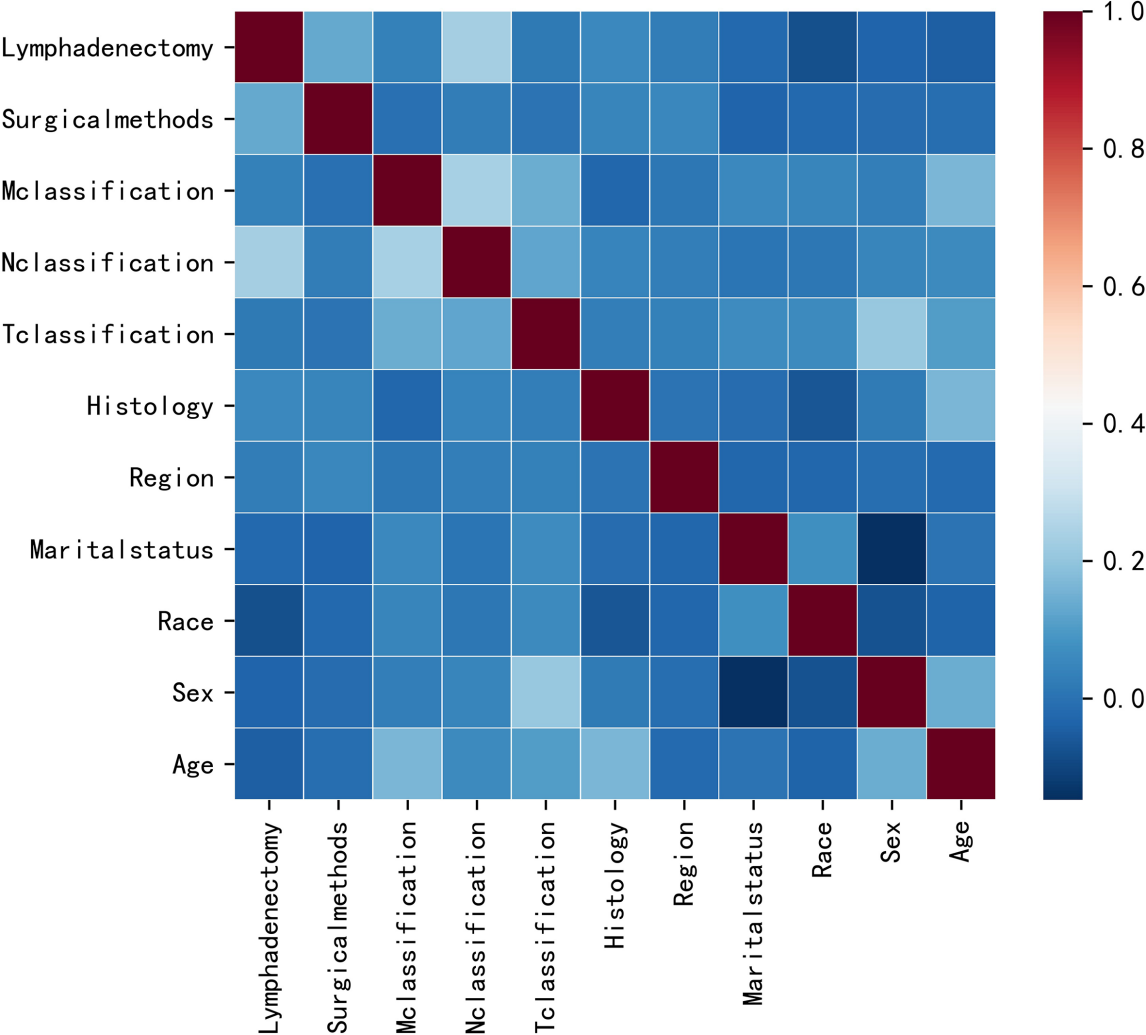
Results of correlation analysis between all variables.

**Figure 3:**
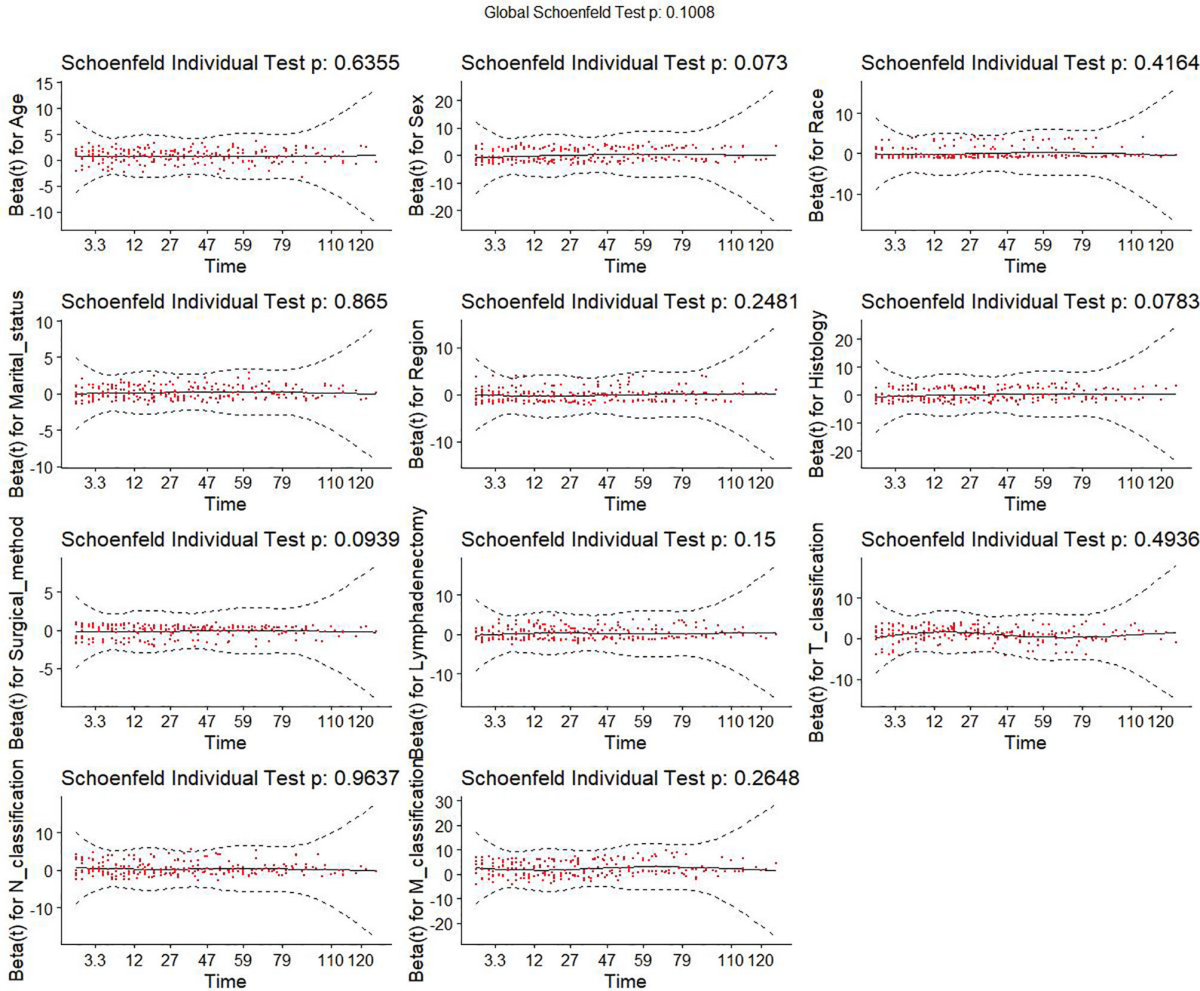
Proportional hazard assumption test of all variables. Y-axis is the beta values for each variable. X-axis is the observed survival time in month. Red dots are the residuals of beta values of different variables. Black solid line is the fitting curve of all residuals. Dashed lines are the lower and upper limits of the 95% confidence interval of all residuals.

This study initially included eleven variables based on professional knowledge. Correlation test was performed among all variables, and the correlation heat map showed that there was no significant correlation among them (**Figure 2**). The VIF of all variables was less than 10, which indicated that there was no multicollinearity among the variables. **Figure 3** indicates the proportional hazard hypothesis test of Cox regression. The results revealed that all residual fitting curves of each variable were close to the level, so it was suitable for the Cox model.

### Univariate and Multivariate Cox Regression Analysis

In univariate analysis, compared with patients who did not undergo surgery, patients who received surgery were closely related to CSS improvements (hazard ratios[HRs]: One-sided thyroid LO plus IO, 0.008[95%CI,0.002-0.027]; TT, 0.041[95%CI,0.028-0.059]; partial thyroidectomy, 0.026[95%CI,0.015-0.046]; S/N TT, 0.048[95%CI,0.027-0.086]; *P* values<0.001 for all comparisons, **Table 2**). Compared with married patients (living with their spouse), patients who were widowed and separated were closely related to CSS deterioration (HRs: Widowed, 5.601[95%CI,4.070-7.708]; separated, 3.839[95%CI,1.875-7.860]; all *P* values<0.001, **Table 2**). The significant variables (*P*<0.05) in the univariate analysis were incorporated into the multivariate analysis. Finally, a total of six variables were included in the Cox regression model (**Table 2**). Among different surgeries, the prognosis of one-sided thyroid LO plus IO (HR, 0.086[95%CI,0.025-0.290], *P*<0.001) was the best, followed by TT (HR, 0.490[95%CI,0.295-0.814], *P*=0.006). Among different marital status, married patients had better prognosis than patients with single (HR,1.686[95%CI,1.146-2.479], *P*=0.008),widowed(HR,1.671[95%CI,1.163-2.402], *P*=0.006), and separated (HR, 4.306[95%CI,2.039-9.093], *P*<0.001) patients.

**Table 2:** Univariate and multivariable analysis of cancer-specific survival in follicular thyroid cancer.

Characteristics	Univariate Survival Analysis		Multivariable Survival Analysis	
	Hazard Ratio (95%CI)	*P* Value	Hazard Ratio (95%CI)	*P* Value
**Age** (y), No.(%)				
<25	1 [Reference]	NA	1 [Reference]	NA
25-40	1.210 (0.262-5.602)	0.807	1.332 (0.284-6.257)	0.716
40-55	2.870 (0.689-11.946)	0.147	2.878 (0.674-12.294)	0.154
55-70	8.018 (1.969-32.653)	0.004	5.269 (1.248-22.253)	0.024
70-85	27.555 (6.798-111.692)	<0.001	14.962 (3.523-63.541)	<0.001
≥85	94.026 (21.872-404.217)	<0.001	32.086 (6.993-147.217)	<0.001
**Sex**, No.(%)				
Female	1.637 (1.264-2.121)	<0.001	1.241 (0.935-1.647)	0.136
**Race**, No.(%)				
White	1 [Reference]	NA		
Black	1.150 (0.777-1.701)	0.485		
Other*	1.407 (0.946-2.095)	0.092		
**Marital status**, No.(%)				
Married	1 [Reference]	NA	1 [Reference]	NA
Single	1.003 (0.703-1.432)	0.986	1.686 (1.146-2.479)	0.008
Divorced	1.499 (0.933-2.409)	0.095	1.396 (0.860-2.265)	0.177
Widowed	5.601 (4.070-7.708)	<0.001	1.671 (1.163-2.402)	0.006
Separated	3.839 (1.875-7.860)	<0.001	4.306 (2.039-9.093)	<0.001
**Region**, No.(%)				
East	1 [Reference]	NA		
Pacific Coast	1.116 (0.84-1.4708)	0.434		
Northern Plains	1.207 (0.765-1.904)	0.419		
Southwest	1.172 (0.668-2.054)	0.580		
**Histology**, No.(%)				
Classic subtype	1 [Reference]	NA	1 [Reference]	NA
HCC/Oxyphilic variant	1.461 (1.127-1.895)	0.004	1.136 (0.861-1.499)	0.366
**Surgical methods**, No.(%)				
No surgery	1 [Reference]	NA	1 [Reference]	NA
Partial thyroidectomy	0.026 (0.015-0.046)	<0.001	0.551 (0.280-1.081)	0.083
One-sided thyroid LO plus IO	0.008 (0.002-0.027)	<0.001	0.086 (0.025-0.290)	<0.001
S/N TT	0.048 (0.027-0.086)	<0.001	0.661 (0.331-1.321)	0.241
TT	0.041 (0.028-0.059)	<0.001	0.490 (0.295-0.814)	0.006
**Lymphadenectomy**, No.(%)				
None	1 [Reference]	NA	1 [Reference]	NA
1 to 3 regional lymph nodes	1.081 (0.780-1.500)	0.639	1.031 (0.720-1.477)	0.868
4 or more regional lymph nodes	2.673 (1.886-3.788)	<0.001	1.366 (0.893-2.090)	0.151
**T classification**, No.(%)				
T1	1 [Reference]	NA	1 [Reference]	NA
T2	1.213 (0.679-2.165)	0.514	1.224 (0.684-2.193)	0.496
T3	4.948 (2.969-8.244)	<0.001	3.146 (1.870-5.291)	<0.001
T4	54.331 (32.134-91.861)	<0.001	10.955 (6.211-19.322)	<0.001
**N classification**, No.(%)				
N0	1 [Reference]	NA	1 [Reference]	NA
N1a	7.716 (4.807-12.384)	<0.001	1.670 (0.954-2.924)	0.072
N1b	22.403 (15.937-31.492)	<0.001	2.248 (1.476-3.424)	<0.001
**M classification**, No.(%)				
M0	1 [Reference]	NA	1 [Reference]	NA
M1	38.357 (29.599-49.706)	<0.001	9.214 (6.669-12.729)	<0.001

*Other include American indian/Alaska native, Asian or Pacific islander; Partial thyroidectomy include lobectomy or lesion resection.

HCC, Hürthle cell carcinoma; CI, Confidence interval; NA, Not applicable; LO plus IO, Lobectomy plus isthmectomy; S/N TT, Subtotal or near total thyroidectomy; TT, Total thyroidectomy.

### Kaplan-Meier Survival Analysis

**Figure 4:**
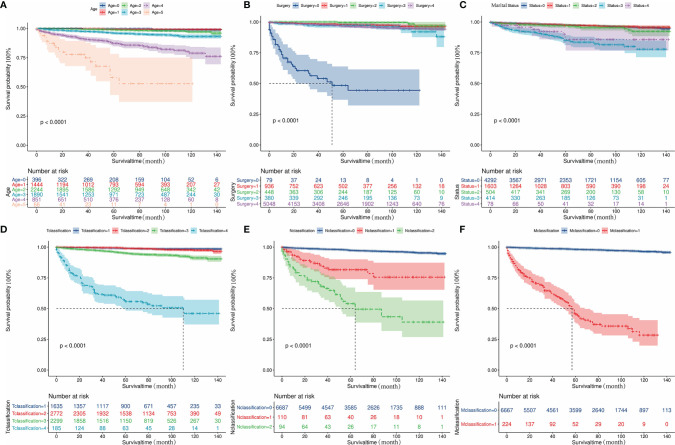
Kaplan-Meier curves depicting cancer-specific survival of important prognostic factors. **(A)** The effect of diagnosis age on the prognosis of patients with FTC. **(B)** The effect of surgical methods on the prognosis of patients with FTC. **(C)** The effect of marital status on the prognosis of patients with FTC. **(D)** The effect of T classification on the prognosis of patients with FTC. **(E)** The effect of N classification on the prognosis of patients with FTC. **(F)** The effect of M classification on the prognosis of patients with FTC. FTC, Follicular thyroid cancer.

**Figure 5:**
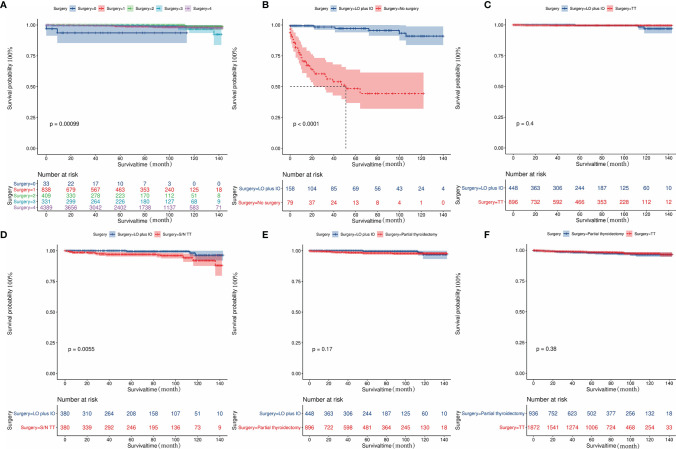
Kaplan-Meier curves depicting cancer-specific survival of different surgical methods in patients. **(A)** indicates the Kaplan-Meier analysis results of different surgical methods in patients with lesions confined to unilateral thyroid capsule and without distant metastasis. **(B-F)** Indicates the Kaplan-Meier analysis results of different surgical methods on tumor prognosis after propensity score matching. The results showed that one-sided LO plus IO, TT, and partial thyroidectomy had no significant difference in long-term prognosis; One-sided thyroid LO+IO had a relatively better prognosis compared with patients without surgery and those who received S/N TT: **(B)** No surgery cases matched to one-sided thyroid LO plus IO (1:2, *P*<0.001). **(C)** TT cases matched to one-sided thyroid LO plus IO (2:1, *P*=0.4); **(D)** S/N TT cases matched to one-sided thyroid LO plus IO (1:1, *P*<0.005); **(E)** Partial thyroidectomy cases matched to one-sided thyroid LO plus IO (2:1, *P*=0.17). **(F)** Partial thyroidectomy cases matched to TT (1:2, *P*=0.38). FTC, Follicular thyroid carcinoma; LO plus IO, Lobectomy plus isthmectomy; S/N TT, Subtotal or near total thyroidectomy; TT, Total thyroidectomy.

The influences of significant prognostic factors on the FTC were shown in the Kaplan-Meier survival plots (**Figures 4A–F**). In addition, Kaplan-Meier survival analysis was also performed for patients whose lesions were only confined to the unilateral thyroid capsule and without distant metastasis (**Figure 5A**). The results showed that compared with patients who did not undergo surgery, patients who underwent surgery had a better prognosis. Propensity scores were used to match one-sided thyroid LO plus IO with other different treatments. The effects of different surgical methods after PSM on FTC prognosis were also described using Kaplan-Meier survival plots (**Figures 5B–F**).The results proved that one-sided LO plus IO, TT, and partial thyroidectomy had no significant differences in long-term prognosis.One-sided thyroid LO plus IO had a relatively better prognosis compared with patients without surgery and those who received S/N TT. The mean survival time and variable settings for each prognostic factor in the Kaplan-Meier curve (**Figures 4**, **5**) were shown in **Table 3**.

**Table 3:** CSS survival time and variable assignment for each significant prognostic factor in Kaplan-Meier analysis results.

Variable assignment	No.(%)	Mean survival time of 143-month, 95%CI	10-year CSS, %	Log Rank	*P*
**Age** (y)				506.119	<0.001
0: <25	396 (5.747)	142.20 (141.09-143.30)	99%		
1: 25-40	1444 (20.955)	142.08 (141.48-142.68)	99%		
2: 40-55	2244 (32.564)	140.79 (140.06-141.53)	97%		
3: 55-70	1890 (27.427)	136.99 (135.66-138.31)	93%		
4: 70-85	851 (12.349)	123.71 (120.23-127.20)	78%		
5: ≥85	66 (0.958)	79.28 (64.32-94.24)	53%		
**Surgical methods**				718.331	<0.001
0: No surgery	79 (1.146)	65.00 (51.25-78.75)	41%		
1: Partial thyroidectomy	936 (13.583)	139.79 (138.40-141.18)	96%		
2: One-sided thyroid LO plus IO	448 (6.501)	141.93 (140.74-143.13)	98%		
3: S/N TT	380 (5.514)	136.34 (133.71-138.96)	91%		
4: TT	5048 (73.255)	138.00 (137.26-138.74)	94%		
**Marital status**				160.665	<0.001
0: Married	4292 (62.284)	139.00 (138.29-139.72)	95%		
1: Single	1603 (23.262)	139.03 (137.84-140.23)	96%		
2: Divorced	504 (7.314)	137.05 (134.52-139.59)	93%		
3: Widowed	414 (6.008)	120.11 (115.34-124.88)	79%		
4: Separated	78 (1.132)	127.33 (117.84-136.82)	84%		
**T classification**				1149.414	<0.001
0: T1	1635 (23.727)	141.47 (140.75-142.19)	–		
1: T2	2772 (40.226)	141.10 (140.48-141.73)	98%		
2: T3	2299 (33.362)	135.51 (134.16-136.86)	97%		
3: T4	185 (2.685)	83.07 (73.65-92.48)	91%		
**N classification**				708.276	<0.001
0: N0	6687 (97.040)	138.93 (138.35-139.52)	95%		
1: N1a	110 (1.596)	115.45 (104.69-126.21)	74%		
2: N1b	94 (1.364)	78.36 (64.99-91.73)	40%		
**M classification**				2038.850	<0.001
0: M0	6667 (96.749)	139.97 (139.46-140.47)	96%		
1: M1	224 (3.251)	66.86 (59.00-74.72)	32%		
**Surgical methods^#^**				18.490	0.001
0: No surgery	33 (0.55)	107.03 (97.69-116.38)	–		
1: Partial thyroidectomy	838 (13.97)	141.74 (140.82-142.67)	99%		
2: One-sided thyroid LO plus IO	409 (6.82)	142.59 (141.79-143.39)	99%		
3: S/N TT	331 (5.52)	140.28 (138.79-141.78)	95%		
4: TT	4389 (73.15)	141.37 (140.91-141.83)	98%		
Surgical method*				68.939	<0.001
0: No surgery	79 (33.33)	65.00 (51.25-78.75)	41%		
1: One-sided thyroid LO plus IO	158 (66.67)	134.02 (129.35-138.69)	91%		
Surgical method*				0.703	0.402
0: TT	896 (66.67)	142.50 (141.93-143.07)	100%		
1: One-sided thyroid LO plus IO	448 (33.33)	141.93 (140.74-143.13)	98%		
Surgical method*				7.716	0.005
0: S/N TT	380 (50.00)	136.34 (133.71-138.96)	91%		
1: One-sided thyroid LO plus IO	380 (50.00)	141.74 (140.34-143.15)	98%		
Surgical method*				1.916	0.166
0: Partial thyroidectomy	896 (66.67)	140.65 (139.42-141.87)	98%		
1: One-sided thyroid LO plus IO	448 (33.33)	141.93 (140.74-143.13)	98%		
Surgical method*				0.772	0.380
0: Partial thyroidectomy	936 (33.33)	139.79 (138.40-141.18)	96%		
1: TT	1872 (66.67)	140.49 (139.61-141.36)	97%		

^#^Indicates the Kaplan-Meier analysis results of different surgical methods in patients with lesions confined to unilateral thyroid capsule and without distant metastasis. *Indicates the Kaplan-Meier analysis results of different surgical methods on tumor prognosis after propensity score matching method. It should be noted that the classification items of some variables cannot estimate the 10-year survival rate of CSS due to the insufficient follow-up time and the lack of end-point events, then we use “-” to indicate.

CSS, Cancer-specific survival; LO plus IO, Lobectomy plus isthmectomy; S/N TT, Subtotal or near total thyroidectomy; TT, Total thyroidectomy; AJCC, American Joint Committee on Cancer.

### Machine Learning Model and AJCC Model

**Figure 6:**
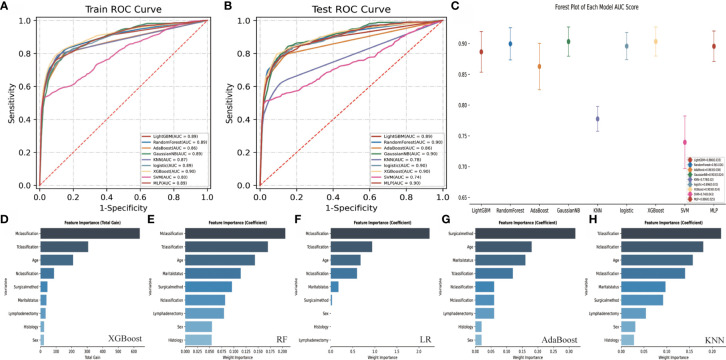
Performance comparison and variable importance ranking of different ML models. **(A)** shows the ROC curve of nine different ML models in the training set. **(B)** shows the ROC curve of nine different ML models in the test set. **(C)** shows the AUROC score forest plot of each model in test set. **(D–H)** show the variable importance ranking of five main ML classifiers. ROC, Receiver operating characteristic; AUROC, Area under the receiver operating characteristic; ML, Machine learning; FTC, Follicular thyroid carcinoma; XGBoost, eXtreme Gradient Boosting; LightGBM, Light Gradient Boosting Machine; LR, Logistic Regression; RF, Random Forests; AdaBoost, Adaptive Boosting; Gaussian NB, Gaussian Naive Bayes; KNN, K-Nearest Neighbor; SVM, Support Vector Machine; MLP, Multi-Layer Perceptron.

**Figure 7:**
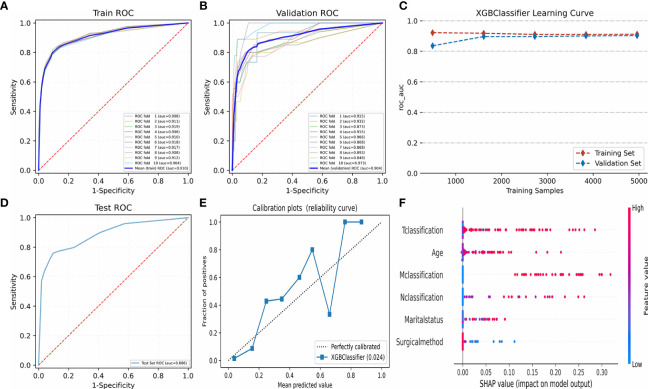
Algorithm optimization and visualization of XGBoost model. **(A–C)** show the fitting optimization process of XGBoost model by 10-fold cross-validation in the training set and verification set. **(D)** shows the AUROC value of XGBoost model in test set. **(E)** shows the calibration plot of XGBoost model. **(F)** shows the SHapley Additive exPlanations of XGBoost model. ROC, Receiver operating characteristic; AUROC, Area under the receiver operating characteristic; XGBoost, eXtreme Gradient Boosting. ROC, Receiver operating characteristic; AUROC, Area under the receiver operating characteristic; XGBoost, eXtreme Gradient Boosting.

**Figure 8:**
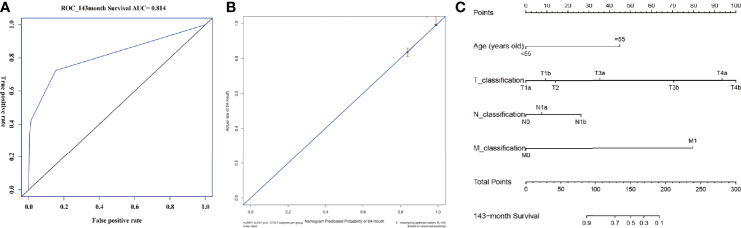
AUROC value, calibration plot and visualization of AJCC model. **(A)** shows the AUROC value of the AJCC model. The y-axis represents the true positive rate of the prognostic prediction, the x-axis represents the false positive rate of the prognostic prediction. The blue solid line represent the predictive performance at 143-month forecast time point. **(B)** shows the calibration plot of the AJCC model. The y-axis represents actual diagnosed cases of FTC, the x-axis represents the nomogram predicated probability. The blue solid line represents a perfect prediction by an ideal model, the red solid line represents the predictive power of the actual model, with the results indicating that a closer fit to the diagonal blue solid line represents a better prediction. **(C)** shows the nomogram of the AJCC model. AUROC, Area under the receiver operating characteristic; AJCC, American Joint Committee on Cancer; FTC: Follicular thyroid carcinoma.

**Table 4** and **Figures 6A–C** display the performance of nine different ML methods. According to the analysis results of the training set and the test set, it was found that the XGBoost model had the best performance. **Figures 6D–H** shows the ranking of variable importance for the five main ML classifiers. All the five ML models showed that age, surgical methods, marital status, T classification, N classification, and M classification were the most important variables affecting the prognosis of FTC. **Figures 7A, B** exhibit the ROC curves of the XGBoost model in the training set and the validation set after 10-fold cross-validation. It can be seen from **Figure 7C** that when the learning curves of the training set and the validation set tend to be the same, the performance of the XGBoost model is the best, and its best AUROC value in the test set is 0.886 (**Figure 7D**). At this time, the parameter settings of the XGBoost model were: Objective: Reg: Logistic, learning_rate: 0.03, max_depth: 3, min_child_weight: 1, reg_lambda: 1. **Figure 7E** shows the calibration plot of XGBoost model, and **Figure 7F** is a SHAP summary of the FTC prognostic model.The higher the SHAP feature value is, the redder the dot color is in the graph, and the lower the SHAP feature value is, the bluer thedot color is in the graph. As shown in the Figure, the larger the value of T classification is, the higher the risk of death in patients of FTC is. A total of four variables were included in the eighth edition of the AJCC cancer staging system, namely age at diagnosis, T classification, N classification, and M classification. The AJCC model was visualized through the nomogram, and the AUROC value of the model was 0.814 (**Figure 8**).

**Table 4:** Comparison prediction performances of different ML models, (Mean ± SD).

Machine learning models		Performance				
		AUROC	Accuracy	Sensitivity	Specificity	NPV
**Training set**	XGBoost^a^	0.905 (0.008)	0.915 (0.013)	0.782 (0.021)	0.914 (0.010)	0.991 (0.001)
	LightGBM	0.891 (0.009)	0.919 (0.034)	0.812 (0.023)	0.866 (0.027)	0.989 (0.002)
	LR	0.891 (0.007)	0.872 (0.028)	0.789 (0.036)	0.871 (0.031)	0.991 (0.001)
	RF	0.895 (0.011)	0.912 0.026	0.800 (0.028)	0.879 (0.028)	0.990 (0.002)
	AdaBoost	0.865 (0.005)	0.946 (0.030)	0.794 (0.030)	0.864 (0.018)	0.986 (0.002)
	Gaussian NB	0.892 (0.006)	0.865 (0.026)	0.790 (0.029)	0.867 (0.027)	0.991 (0.001)
	KNN	0.870 (0.015)	0.971 (0.002)	0.793 (0.034)	0.909 (0.027)	0.982 (0.001)
	SVM	0.797 (0.014)	0.940 (0.041)	0.567 (0.042)	0.951 (0.043)	0.984 (0.001)
	MLP	0.894 (0.016)	0.903 (0.024)	0.765 (0.042)	0.905 (0.024)	0.990 (0.001)
**Test set**	XGBoost^b^	0.904 (0.024)	0.906 (0.025)	0.809 (0.069)	0.903 (0.023)	0.991 (0.003)
	LightGBM	0.887 (0.033)	0.920 (0.046)	0.804 (0.068)	0.883 (0.043)	0.989 (0.002)
	LR	0.897 (0.022)	0.863 (0.036)	0.833 (0.052)	0.862 (0.039)	0.991 (0.003)
	RF	0.900 (0.026)	0.908 (0.047)	0.812 (0.063)	0.887 (0.041)	0.990 (0.002)
	AdaBoost	0.863 (0.038)	0.959 (0.007)	0.772 (0.064)	0.889 (0.046)	0.985 (0.003)
	Gaussian NB	0.904 (0.024)	0.888 (0.029)	0.830 (0.057)	0.885 (0.033)	0.991 (0.003)
	KNN	0.778 (0.020)	0.965 (0.005)	0.619 (0.047)	0.905 (0.032)	0.978 (0.004)
	SVM	0.740 (0.043)	0.954 (0.024)	0.522 (0.091)	0.970 (0.029)	0.981 (0.004)
	MLP	0.896 (0.025)	0.893 (0.044)	0.797 (0.039)	0.893 (0.044)	0.990 (0.003)

^**a**^ indicates that the best performance of the ML classifiers in the training set is XGBoost (ranked mainly according to AUROC value); ^**b**^ indicates that the best performance of the ML classifiers in the test set is XGBoost (ranked mainly according to AUROC and accuracy value).

AUROC, Area under the receiver operating characteristic; NPV, Negative predictive value; XGBoost, eXtreme Gradient Boosting; LightGBM, Light Gradient Boosting Machine; LR, Logistic Regression; RF, Random Forests; AdaBoost, Adaptive Boosting; Gaussian NB, Gaussian Naive Bayes; KNN, K-Nearest Neighbor; SVM, Support Vector Machine; MLP, Multi-Layer Perceptron.

## Discussion

In this study, it was observed that treatment methods (different surgical methods or active surveillance) and marital status were important prognostic factors related to CSS based on univariate and multivariate Cox regression model. Our results reshaped the traditional view that TT was the standard for treating FTC. The results of multivariate Cox regression were used to construct ML models for FTC patients. The variables in the ML models include age, surgical methods, marital status, T classification, N classification and M classification. As far as we know, this is the first article that uses different ML methods and AJCC cancer staging system to predict the long-term survival of FTC. Our study showed that the XGBoost model appears to have better predictive accuracy than the traditional AJCC cancer staging system.

The analysis of prognostic factors of TC is necessary, especially for FTC patients with relatively high mortality and prone to distant metastases. Unfortunately, due to the lack of clinical data (Because compared with PTC, the prevalence and awareness of FTC is lower) and the low incidence of end-point events, it is difficult to establish prognostic model for CSS of FTC. Secondly, most prediction models or staging systems currently used in clinical practice are for individuals with DTC (including PTC and FTC), medullary cancer and undifferentiated cancer, rather than FTC patients. Thirdly, the predictors of these models mainly include age at diagnosis, tumor size, lymph nodes and distant metastasis, while ignoring other common factors that may affect the prognosis of FTC, such as sociological factors and surgical methods.Therefore, we hold the view that establishing a complete prognostic model for FTC patients has important clinical significance.

As a classic statistical method that is often used to develop clinical prognostic models, Cox regression belongs to regression analysis, which predicts event probability by selecting and using a small number of variables. Most importantly, Cox regression considers the time of the event in its prediction process, and the model performance is better. Meanwhile, it can express the patient’s predictive effect in a simple and easy-to-interpret form (HR), and visualize it in the form of a nomogram. Therefore, Cox regression was used as a method of variable screening and a modeling tool for traditional cancer staging systems. In clinical practice, the current eighth edition AJCC cancer staging system is a widely used and accepted model (22). It is worth noting that in this staging system, FTC is usually studied in combination with PTC (ie, DTC). Therefore, the clinical prognosis model of FTC was constructed based on the eighth edition of the AJCC staging system. With the continuous development of the precision medicine field, people have put forward higher requirements for the accuracy and applicability of various models. Some studies have proved that ML has stronger data processing and knowledge acquisition capabilities compared with traditional statistics. Obviously, this innovative method is an important tool in the field of precision medicine, and helps to choose the best diagnosis and treatment strategy.

In this study, nine different novel ML algorithms were applied to construct the prognostic model of FTC. According to some research, the XGBoost model had better predictive performance than other predictive models, no matter in the training set or the test set. Most importantly, it seems to have better prediction accuracy than the traditional AJCC model. XGBoost is a boosted tree model. The applied algorithm is based on the improvement of GBDT. It can be used to solve classification problems as well as regression problems. In recent years, more and more clinical studies have used the XGBoost algorithm for disease screening, prevention and diagnosis, with positive results. A study from Wu et al. (45) revealed that in determining the clinical prognosis of young hypertensive patients, the XGBoost model was comparable to the Cox regression method and better than the recalibrated Framingham Risk Score model. Hou et al. (46) used XGBoost to develop an ML method to predict the 30-day mortality of sepsis patients. This studies illustrated that the XGBoost model has the best predictive value (AUC,0.857[95%CI,0.839-0.876]) compared with the traditional LR model (AUC,0.819[95%CI, 0.800-0.838]) and simplified acute physiological score II (SAPS II) score prediction model (AUC,0.797[95%CI, 0.781-0.813]). In addition, the research conducted by Zheng et al. (47) also demonstrated that the XGBoost model based on real-world evidence had good predictive performance in predicting the blood concentration of tacrolimus, which could provide guidance for the adjustment of the plan in clinical practice. Five commonly used ML algorithms were used to rank FTC’s risk factors in importance. The research results showed that age, surgical methods, marital status, T classification, N classification and M classification were important variables that affect the prognosis of FTC, which was consistent with the analysis results of multivariate Cox regression. It is worth noting that the risk factors of age, T classification, N classification and M classification have been fully discussed in previous observational studies (48, 49), but the impacts of different surgical methods and sociological factors (marital status) on the prognosis of FTC are still unknown. So, we conducted a detailed analysis on these variables.

Surgery is the main way to treat TC, but the choice of surgical method is still controversial (9, 10, 50). Since FTC is more aggressive than PTC, early treatment is essential to improve the prognosis of FTC patients. Our research confirmed that whether it was univariate or multivariate analysis, surgical methods had high HR values, which suggested that surgeries were important prognostic indicators of FTC. FTC is mostly unilateral lesions, and TT can lead to permanent hypothyroidism or even hypoparathyroidism, which seriously affects the quality of patient’s life. Therefore, some scholars suggested that patients with FTC with a single lesion on one side and no high-risk factors can perform one-sided thyroid LO plus IO (51, 52). Some scholars also argued that as long as the tumor was confined to one lobe, TT should also be performed (51). For this reason, we performed Kaplan-Meier survival analysis for 6000 patients whose lesions were only confined to the unilateral capsule and no distant metastasis. The results revealed that one-sided thyroid LO plus IO was still the best treatment, followed by local surgical excision, TT and S/N TT (Log Rank=18.49, *P*=0.001). In order to further control the confounding, a PSM analysis was conducted, and the results proved that one-sided thyroid LO plus IO, TT, and local surgical excision had no significant differences in long-term prognosis. It should be noted that the type of initial surgical intervention should consider all risk factors such as tumor size, lymph node metastasis and distant metastasis, which is the primary factor in determining the type of treatment. The subjects included in this study were mainly FTC patients with early non-lymph node and distant metastases. Compared with TT, one-sided thyroid LO plus IO or partial thyroidectomy also can achieve a good prognosis, which is of positive significance for guiding clinical practice.

In recent years, some studies have revealed that marital status is closely related to the prognosis of TC (11, 12, 53) and married TC patients have a significant survival advantage. A study from 126,160 patients with all types of TC showed that widowed or divorced patients were closely related to poor CSS and overall survival (OS) (11). Shi et al. explored 61077 DTC patients and found that widowed patients had a higher tumor mortality in DTC (12). A study from Roche et al. indicated that for MTC patients, being married had a protective effect on treatment and overall 5-year survival, but had no effects on CSS (53). In this study, the impacts of marital status on the prognosis of FTC were evaluated. The results found that married people had a better prognosis than single, widowed, and separated patients. More and more studies have shown that a good marital status plays a positive role in the prognosis of tumors, such as bladder cancer (54), oral cancer (55), colorectal cancer (56, 57), chordoma (58), head and neck cancer (59, 60), renal cell carcinoma (61), and so on. The generally accepted explanation for the lower cancer death rate among married people is related to a better socioeconomic status, which is assumed to buffer the impacts of stressful events (62). It is well known that TC is an endocrine-related disease, and mood changes and mental health are closely related to the prognosis of TC. Therefore, we think that providing effective psychological counseling and social support for unmarried, widowed, and separated patients has positive effects on the improvement of the prognosis.

This study also has the following limitations. Firstly, even if the internal differences in baseline characteristics were adjusted through multivariate Cox regression and PSM, these differences still existed to a large extent. Due to the limitation of follow-up time, the longest predicted time point was 143 months. We know that TC has a good prognosis and a high 10-year survival rate, so in future studies, a longer follow-up period should be included. Secondly, the population of this study was mainly from Western countries. Although it included different races, the number of Asians was small. In future research, model verification should be conducted through external populations. Thirdly, we classified the TNM staging of FTC patients with reference to the eighth edition of the AJCC cancer staging guidelines. Owing to the limitations of the database itself, there may be minor discrepancies in tumor staging, which needs to be further improved in future clinical studies.

## Conclusions

In summary, the impacts of different surgical methods and marital status on the long-term prognosis of FTC were described. Our studies have proved that for most patients with non-lymph node and distant metastases, one-sided thyroid LO plus IO has a better long-term prognosis. In addition, active and effective social support and companionship can improve the CSS of FTC patients. The XGBoost model can better communicate the prognosis and ultimately promote patient decision-making based on new risk factors.

## Data Availability Statement

The original contributions presented in the study are included in the article/supplementary material. Further inquiries can be directed to the corresponding author.

## Author Contributions

GC had full access to all of the data in the study and takes responsibility for the integrity of the data and the accuracy of the data analysis; Concept and design, YM, GC, JL, YH, LX, WL, JW, HH, and LL; Acquisition, analysis, or interpretation of data, YM, YH, and LX; Drafting of the manuscript, YM, YH, LX, and GC; Critical revision of the manuscript for important intellectual content, YM, WL, JW, GC, JL, HH, and LL; Statistical analysis, YM, YH, and LX; Supervision, GC; All authors contributed to the article and approved the submitted version.

## Conflict of Interest

The authors declare that the research was conducted in the absence of any commercial or financial relationships that could be construed as a potential conflict of interest.

## Publisher’s Note

All claims expressed in this article are solely those of the authors and do not necessarily represent those of their affiliated organizations, or those of the publisher, the editors and the reviewers. Any product that may be evaluated in this article, or claim that may be made by its manufacturer, is not guaranteed or endorsed by the publisher.

## References

[1] La Vecchia C Malvezzi M Bosetti C Garavello W Bertuccio P Levi F. Thyroid Cancer Mortality and Incidence: A Global Overview. Int J Cancer (2015) 136(9):2187–95. doi: 10.1002/ijc.29251 25284703

[2] Siegel R Miller K Jemal A. Cancer Statistics, 2020. CA Cancer J Clin (2020) 70(1):7–30. doi: 10.3322/CAAC.21590 31912902

[3] Lim H Devesa SS Sosa JA Check D Kitahara CM. Trends in Thyroid Cancer Incidence and Mortality in the United States, 1974-2013. JAMA (2017) 317(13):1338–48. doi: 10.1001/jama.2017.2719 PMC8216772 28362912

[4] Urken M. Prognosis and Management of Invasive Well-Differentiated Thyroid Cancer. Otolaryngol Clin North Am (2010) 43(2):301–28. doi: 10.1016/j.otc.2010.02.002 20510716

[5] Rodríguez-Cuevas S Labastida-Almendaro S Cortés-Arroyo H López-Garza J Barroso-Bravo S. Multifactorial Analysis of Survival and Recurrences in Differentiated Thyroid Cancer. Comparative Evaluation of Usefulness of AGES, MACIS, and Risk Group Scores in Mexican Population. J Exp Clin Cancer Res (2002) 21(1):79–86. 12071534

[6] Lundgren CI Hall P Dickman PW Zedenius J. Clinically Significant Prognostic Factors for Differentiated Thyroid Carcinoma: A Population-Based, Nested Case-Control Study. Cancer (2006) 106(3):524–31. doi: 10.1002/cncr.21653 16369995

[7] Hassan A Razi M Riaz S Khalid M Nawaz MK Syed AA. Survival Analysis of Papillary Thyroid Carcinoma in Relation to Stage and Recurrence Risk: A 20-Year Experience in Pakistan. Clin Nucl Med (2016) 41(8):606–13. doi: 10.1097/RLU.0000000000001237 27124680

[8] O'Neill CJ Vaughan L Learoyd DL Sidhu SB Delbridge LW Sywak MS. Management of Follicular Thyroid Carcinoma Should be Individualised Based on Degree of Capsular and Vascular Invasion. Eur J Surg Oncol (2011) 37(2):181–5. doi: 10.1016/j.ejso.2010.11.005 21144693

[9] Nixon IJ Ganly I Patel SG Palmer FL Whitcher MM Tuttle RM. Thyroid Lobectomy for Treatment of Well Differentiated Intrathyroid Malignancy. Surgery (2012) 151(4):571–9. doi: 10.1016/j.surg.2011.08.016 22001636

[10] Bilimoria KY Bentrem DJ Ko CY Stewart AK Winchester DP Talamonti MS. Extent of Surgery Affects Survival for Papillary Thyroid Cancer. Ann Surg (2007) 246(3):375–81. doi: 10.1097/SLA.0b013e31814697d9 PMC1959355 17717441

[11] Li Y Huang D Wang B Mao W Chen X Dong P. Socioeconomic Factors are Associated With the Prognosis of Thyroid Cancer. J Cancer (2021) 12(9):2507–12. doi: 10.7150/jca.52329 PMC8040706 33854612

[12] Shi RL Qu N Lu ZW Liao T Gao Y Ji QH. The Impact of Marital Status at Diagnosis on Cancer Survival in Patients With Differentiated Thyroid Cancer. Cancer Med (2016) 5(8):2145–54. doi: 10.1002/cam4.778 PMC4898978 27264532

[13] Hsieh MH Sun LM Lin CL Hsieh MJ Hsu CY Kao CH. The Performance of Different Artificial Intelligence Models in Predicting Breast Cancer Among Individuals Having Type 2 Diabetes Mellitus. Cancers (Basel) (2019) 11(11):1751. doi: 10.3390/cancers11111751 PMC6895886 31717292

[14] Rau HH Hsu CY Lin YA Atique S Fuad A Wei LM. Development of a Web-Based Liver Cancer Prediction Model for Type II Diabetes Patients by Using an Artificial Neural Network. Comput Methods Programs BioMed (2016) 125:58–65. doi: 10.1016/j.cmpb.2015.11.009 26701199

[15] Hsieh MH Sun LM Lin CL Hsieh MJ Sun K Hsu CY. Development of a Prediction Model for Colorectal Cancer Among Patients With Type 2 Diabetes Mellitus Using a Deep Neural Network. J Clin Med (2018) 7(9):277. doi: 10.3390/jcm7090277 PMC6162847 30213141

[16] Yoo TK Kim SK Kim DW Choi JY Lee WH Oh E. Osteoporosis Risk Prediction for Bone Mineral Density Assessment of Postmenopausal Women Using Machine Learning. Yonsei Med J (2013) 54(6):1321–30. doi: 10.3349/ymj.2013.54.6.1321 PMC3809875 24142634

[17] Weng SF Reps J Kai J Garibaldi JM Qureshi N. Can Machine-Learning Improve Cardiovascular Risk Prediction Using Routine Clinical Data? PloS One (2017) 12(4):e0174944. doi: 10.1371/journal.pone.0174944 28376093 PMC5380334

[18] Senders JT Staples PC Karhade AV Zaki MM Gormley WB Broekman MLD. Machine Learning and Neurosurgical Outcome Prediction: A Systematic Review. World Neurosurg (2018) 109:476–486.e1. doi: 10.1016/j.wneu.2017.09.149 28986230

[19] Taylor RA Pare JR Venkatesh AK Mowafi H Melnick ER Fleischman W. Prediction of In-Hospital Mortality in Emergency Department Patients With Sepsis: A Local Big Data-Driven, Machine Learning Approach. Acad Emerg Med (2016) 23(3):269–78. doi: 10.1111/acem.12876 PMC5884101 26679719

[20] Singal AG Mukherjee A Elmunzer BJ Higgins PD Lok AS Zhu J. Machine Learning Algorithms Outperform Conventional Regression Models in Predicting Development of Hepatocellular Carcinoma. Am J Gastroenterol (2013) 108(11):1723–30. doi: 10.1038/ajg.2013.332 PMC4610387 24169273

[21] Churpek MM Yuen TC Winslow C Meltzer DO Kattan MW Edelson DP. Multicenter Comparison of Machine Learning Methods and Conventional Regression for Predicting Clinical Deterioration on the Wards. Crit Care Med (2016) 44(2):368–74. doi: 10.1097/CCM.0000000000001571 PMC4736499 26771782

[22] Perrier ND Brierley JD Tuttle RM. Differentiated and Anaplastic Thyroid Carcinoma: Major Changes in the American Joint Committee on Cancer Eighth Edition Cancer Staging Manual. CA Cancer J Clin (2018) 68(1):55–63. doi: 10.3322/caac.21439 29092098 PMC5766386

[23] Grambsch PM Therneau TM. Proportional Hazards Tests and Diagnostics Based on Weighted Residuals. Biometrika (1994) 81(3):515–26. doi: 10.1093/biomet/81.3.515

[24] Hu CA Chen CM Fang YC Liang SJ Wang HC Fang WF. Using a Machine Learning Approach to Predict Mortality in Critically Ill Influenza Patients: A Cross-Sectional Retrospective Multicentre Study in Taiwan. BMJ Open (2020) 10(2):e033898. doi: 10.1136/bmjopen-2019-033898 PMC7045134 32102816

[25] Ogami C Tsuji Y Seki H Kawano H To H Matsumoto Y. An Artificial Neural Network-Pharmacokinetic Model and its Interpretation Using Shapley Additive Explanations. CPT Pharmacometrics Syst Pharmacol (2021) 10(7):760–8. doi: 10.1002/psp4.12643 PMC8302242 33955705

[26] Xu Y Yang X Huang H Peng C Ge Y Wu H. Extreme Gradient Boosting Model Has a Better Performance in Predicting the Risk of 90-Day Readmissions in Patients With Ischaemic Stroke. J Stroke Cerebrovasc Dis (2019) 28(12):104441. doi: 10.1016/j.jstrokecerebrovasdis.2019.104441 31627995

[27] Yan J Xu Y Cheng Q Jiang S Wang Q Xiao Y. LightGBM: Accelerated Genomically Designed Crop Breeding Through Ensemble Learning. Genome Biol (2021) 22(1):271. doi: 10.1186/s13059-021-02492-y 34544450 PMC8451137

[28] Sekhar CR Minal Madhu E. Mode Choice Analysis Using Random Forrest Decision Trees. Transp Res Proc (2016) 17:644–52. doi: 10.1016/j.trpro.2016.11.119

[29] Rigatti SJ. Random Forest. J Insur Med (2017) 47(1):31–9. doi: 10.17849/insm-47-01-31-39.1 28836909

[30] Huang HJ Hsu CN. Bayesian Classification for Data From the Same Unknown Class. IEEE Trans Syst Man Cybern B Cybern (2002) 32(2):137–45. doi: 10.1109/3477.990870 18238113

[31] Maheswari S Pitchai R. Heart Disease Prediction System Using Decision Tree and Naive Bayes Algorithm. Curr Med Imaging Rev (2019) 15(8):712–7. doi: 10.2174/1573405614666180322141259 32008540

[32] LaValley MP. Logistic Regression. Circulation (2008) 117(18):2395–9. doi: 10.1161/CIRCULATIONAHA.106.682658 18458181

[33] Domínguez-Almendros S Benítez-Parejo N Gonzalez-Ramirez AR. Logistic Regression Models. Allergol Immunopathol (Madr) (2011) 39(5):295–305. doi: 10.1016/j.aller.2011.05.002 21820234

[34] Schober P Vetter TR. Logistic Regression in Medical Research. Anesth Analg (2021) 132(2):365–6. doi: 10.1213/ANE.0000000000005247 PMC7785709 33449558

[35] Guo HG Fang M. Application of AdaBoost Method in IDS. Comput Appl (2005) 25(01):144–6. doi: 10.3724/SP.J.1087.2005.0144

[36] Borghi PH Zakordonets O Teixeira JP. A COVID-19 Time Series Forecasting Model Based on MLP ANN. Proc Comput Sci (2021) 181:940–7. doi: 10.1016/j.procs.2021.01.250 PMC8076817 33936325

[37] Kim G Kim Y Lim H Kim H. An MLP-Based Feature Subset Selection for HIV-1 Protease Cleavage Site Analysis. Artif Intell Med (2010) 48(2-3):83–9. doi: 10.1016/j.artmed.2009.07.010 19945261

[38] Zhang Z. Introduction to Machine Learning: K-Nearest Neighbors. Ann Transl Med (2016) 4(11):218. doi: 10.21037/atm.2016.03.37 27386492 PMC4916348

[39] Noble WS. What Is a Support Vector Machine? Nat Biotechnol (2006) 24(12):1565– 1567. doi: 10.1038/nbt1206-1565 17160063

[40] Henneges C Bullinger D Fux R Friese N Seeger H Neubauer H. Prediction of Breast Cancer by Profiling of Urinary RNA Metabolites Using Support Vector Machine-Based Feature Selection. BMC Cancer (2009) 9:104. doi: 10.1186/1471-2407-9-104 19344524 PMC2680413

[41] Huang S Cai N Pacheco PP Narrandes S Wang Y Xu W. Applications of Support Vector Machine (SVM) Learning in Cancer Genomics. Cancer Genomics Proteomics (2018) 15(1):41–51. doi: 10.21873/cgp.20063 29275361 PMC5822181

[42] Han M Dai J Zhang Y Lin Q Jiang M Xu X. Support Vector Machines Coupled With Proteomics Approaches for Detecting Biomarkers Predicting Chemotherapy Resistance in Small Cell Lung Cancer. Oncol Rep (2012) 28(6):2233–8. doi: 10.3892/or.2012.2037 22992788

[43] Hazai E Hazai I Ragueneau-Majlessi I Chung SP Bikadi Z Mao Q. Predicting Substrates of the Human Breast Cancer Resistance Protein Using a Support Vector Machine Method. BMC Bioinform (2013) 14:130. doi: 10.1186/1471-2105-14-130 PMC3641962 23586520

[44] Kane LT Fang T Galetta MS Goyal DKC Nicholson KJ Kepler CK. Propensity Score Matching: A Statistical Method. Clin Spine Surg (2020) 33(3):120–2. doi: 10.1097/BSD.0000000000000932 31913173

[45] Wu X Yuan X Wang W Liu K Qin Y Sun X. Value of a Machine Learning Approach for Predicting Clinical Outcomes in Young Patients With Hypertension. Hypertension (2020) 75(5):1271–8. doi: 10.1161/HYPERTENSIONAHA.119.13404 32172622

[46] Hou N Li M He L Xie B Wang L Zhang R. Predicting 30-Days Mortality for MIMIC-III Patients With Sepsis-3: A Machine Learning Approach Using XGboost. J Transl Med (2020) 18(1):462. doi: 10.1186/s12967-020-02620-5 33287854 PMC7720497

[47] Zheng P Yu Z Li L Liu S Lou Y Hao X. Predicting Blood Concentration of Tacrolimus in Patients With Autoimmune Diseases Using Machine Learning Techniques Based on Real-World Evidence. Front Pharmacol (2021) 12:727245. doi: 10.3389/fphar.2021.727245 34630104 PMC8497784

[48] Zhang R Xu M Liu X Wang M Jia Q Wang S. Establishment and Validation of a Nomogram Model for Predicting the Survival Probability of Differentiated Thyroid Carcinoma Patients: A Comparison With the Eighth Edition AJCC Cancer Staging System. Endocrine (2021) 74(1):108–19. doi: 10.1007/s12020-021-02717-x 33822318

[49] Kim M Kim YN Kim WG Park S Kwon H Jeon MJ. Optimal Cut-Off Age in the TNM Staging System of Differentiated Thyroid Cancer: Is 55 Years Better Than 45 Years? Clin Endocrinol (Oxf) (2017) 86(3):438–43. doi: 10.1111/cen.13254 27731521

[50] van Gerwen M Alsen M Lee E Sinclair C Genden E Taioli E. Recurrence-Free Survival After Total Thyroidectomy and Lobectomy in Patients With Papillary Thyroid Microcarcinoma. J Endocrinol Invest (2021) 44(4):725–34. doi: 10.1007/s40618-020-01342-1 32651895

[51] Yin D Tang Y. Hotspots of Diagnosis and Treatment of Follicular Thyroid Carcinoma. J Xi’an Jiaotong Univ (Med Sci) (2019) 40(3):339–42. doi: 10.7652/jdyxb201903001

[52] National Health Commission of the People’s Republic of China. Guidelines for Diagnosis and Treatment of Thyroid Cancer (2018 Edition). Chin Arch Gen Surg (Electronic Edition) (2019) 13(1):1–15. doi: 10.3877/cma.j.issn

[53] Roche AM Fedewa SA Chen AY. Association of Socioeconomic Status and Race/Ethnicity With Treatment and Survival in Patients With Medullary Thyroid Cancer. JAMA Otolaryngol Head Neck Surg (2016) 142(8):763–71. doi: 10.1001/jamaoto.2016.1051 27254481

[54] Niu Q Lu Y Wu Y Xu S Shi Q Huang T. The Effect of Marital Status on the Survival of Patients With Bladder Urothelial Carcinoma: A SEER Database Analysis. Medicine (Baltimore) (2018) 97(29):e11378. doi: 10.1097/MD.0000000000011378 30024509 PMC6086512

[55] Liao PH Lee CC. The Influence of Marital Status on Survival for Patients Aged 65 Years and Younger With Oral Cavity Cancer. Auris Nasus Larynx (2018) 45(6):1227–32. doi: 10.1016/j.anl.2018.03.007 29685504

[56] Yang CC Cheng LC Lin YW Wang SC Ke TM Huang CI. The Impact of Marital Status on Survival in Patients With Surgically Treated Colon Cancer. Medicine (Baltimore) (2019) 98(11):e14856. doi: 10.1097/MD.0000000000014856 30882684 PMC6426559

[57] Feng Y Dai W Li Y Mo S Li Q Cai S. The Effect of Marital Status by Age on Patients With Colorectal Cancer Over the Past Decades: A SEER-Based Analysis. Int J Colorectal Dis (2018) 33(8):1001–10. doi: 10.1007/s00384-018-3017-7 29546559

[58] Tang C Wang R Lu Q Wang S Jia G Cao P. Influence of Marital Status on Overall Survival in Adult Patients With Chordoma: A SEER-Based Study. J Orthop Surg Res (2020) 15(1):278. doi: 10.1186/s13018-020-01803-6 32703313 PMC7376721

[59] Osazuwa-Peters N Christopher KM Cass LM Massa ST Hussaini AS Behera A. What's Love Got to do With it? Marital Status and Survival of Head and Neck Cancer. Eur J Cancer Care (Engl) (2019) 28(4):e13022. doi: 10.1111/ecc.13022 30784126

[60] Simpson MC Challapalli SD Cass LM Zahirsha ZS Adjei Boakye E Massa ST. Impact of Gender on the Association Between Marital Status and Head and Neck Cancer Outcomes. Oral Oncol (2019) 89:48–55. doi: 10.1016/j.oraloncology.2018.12.009 30732958

[61] Marchioni M Martel T Bandini M Pompe RS Tian Z Kapoor A. Marital Status and Gender Affect Stage, Tumor Grade, Treatment Type and Cancer Specific Mortality in T_1_-_2_ N_0_ M_0_ Renal Cell Carcinoma. World J Urol (2017) 35(12):1899–905. doi: 10.1007/s00345-017-2082-9 28849260

[62] Giese-Davis J Waller A Carlson LE Groff S Zhong L Neri E. Screening for Distress, the 6th Vital Sign: Common Problems in Cancer Outpatients Over One Year in Usual Care: Associations With Marital Status, Sex, and Age. BMC Cancer (2012) 12:441. doi: 10.1186/1471-2407-12-441 23031647 PMC3528655

